# Machine Learning for Assessment of Coronary Artery Disease in Cardiac CT: A Survey

**DOI:** 10.3389/fcvm.2019.00172

**Published:** 2019-11-26

**Authors:** Nils Hampe, Jelmer M. Wolterink, Sanne G. M. van Velzen, Tim Leiner, Ivana Išgum

**Affiliations:** ^1^Department of Biomedical Engineering and Physics, Amsterdam University Medical Center, University of Amsterdam, Amsterdam, Netherlands; ^2^Amsterdam Cardiovascular Sciences, Amsterdam University Medical Center, University of Amsterdam, Amsterdam, Netherlands; ^3^Image Sciences Institute, University Medical Center Utrecht, Utrecht, Netherlands; ^4^Department of Radiology, University Medical Center Utrecht, Utrecht, Netherlands; ^5^Department of Radiology and Nuclear Medicine, Amsterdam University Medical Center, University of Amsterdam, Amsterdam, Netherlands

**Keywords:** machine learning, coronary artery disease, atherosclerotic plaque, coronary artery stenosis, cardiac CT

## Abstract

Cardiac computed tomography (CT) allows rapid visualization of the heart and coronary arteries with high spatial resolution. However, analysis of cardiac CT scans for manifestation of coronary artery disease is time-consuming and challenging. Machine learning (ML) approaches have the potential to address these challenges with high accuracy and consistent performance. In this mini review, we present a survey of the literature on ML-based analysis of coronary artery disease in cardiac CT. We summarize ML methods for detection and characterization of atherosclerotic plaque as well as anatomically and functionally significant coronary artery stenosis.

## 1. Introduction

Diagnosis and monitoring of coronary artery disease (CAD) is increasingly based on non-invasive imaging with computed tomography (CT), allowing excellent visualization of the coronary arteries with high spatial resolution. Cardiac CT exams consist of hundreds of slices and the number of cardiac CT studies has been steadily increasing (1). This has led to an increased workload for medical professionals, which in combination with shortages of trained cardiac imagers (2) might lead to cardiac CT underuse in the clinic. Machine learning (ML) could offer a way to address these challenges and facilitate automatic cardiac CT analysis with consistent and accurate results. Furthermore, ML algorithms might enable an increased range of secondary diagnoses.

This survey provides an overview of ML algorithms for detection, characterization, and quantification of CAD in cardiac CT. We searched PubMed for articles related to ML-based assessment of CAD in cardiac CT published within the last 10 years (search strategy in Supplementary Materials) which led to inclusion of 59 studies. The structure of this survey is as follows. We provide a brief primer on ML in section 2. Applications of ML for automatic detection and characterization of atherosclerotic plaque are summarized in section 3. Studies focusing on ML for anatomical and functional evaluation of luminal stenosis are summarized in section 4. Finally, section 5 provides a discussion of outstanding challenges for transfer of ML algorithms into the clinic.

## 2. Machine Learning

Machine learning comes in many flavors, but most applications in cardiac CT use supervised learning. In supervised learning, a model is optimized to provide the correct labels as defined by the reference standard during *training*, and predict a label to new and unseen samples during *testing*.

Each sample can be described based on characteristics or *features*. Among the simplest ML algorithms are k-nearest neighbor (kNN) classifiers, which look for training samples with similar feature values to a test sample, and assign the test sample to the majority class among these training samples. Linear classifier (LC) models like support vector machines (SVM) aim to find a linear combination of features to separate samples in different classes. Alternatively, samples can be separated by thresholding feature values along a single axis. This is unlikely to lead to highly accurate classifiers, but by consecutively applying thresholds, a decision tree model can be built for more accurate classification.

ML performance can often be improved by combining predictions of multiple models. Ensembles (E) combine predictions of multiple simultaneously executed models, e.g., by averaging predictions of decision trees in a random forest (RF). In boosting (BO), models are applied consecutively and each model is trained to correct errors of its predecessors. Finally, artificial neural networks (ANNs) transform samples into targets through layers of trainable neurons, which are loosely based on biological neurons. While ANNs have been around since the 1950s, it has recently become possible to train networks that have many layers, i.e., *deep learning*. The success of deep learning in medical image analysis has been to a large extent due to the inclusion of trainable image filters in so-called convolutional neural networks (CNNs), which can be trained to extract valuable features from raw image data (3). For a more in-depth introduction to ML and deep learning, please refer to Jordan and Mitchell (4).

## 3. Atherosclerotic Plaque Detection, Characterization, and Quantification

CT offers a non-invasive alternative to e.g., catheter-guided X-ray angiography, optical coherence tomography, and intravascular ultrasound (IVUS) for atherosclerotic plaque visualization. Characterization and quantification of plaque in CT provide insight in different stages of CAD (5). In this section, we survey analysis of methods for calcified plaque (section 3.1) and non-calcified and mixed plaque (section 3.2). Reviewed papers are listed in Table 1.

**Table 1:** Publications related to analysis of (A) calcified and (B) non-calcified and mixed atherosclerotic plaque.

**(A) Calcified plaque**	**CSCT**	**Chest CT**	**CCTA**	**Training**	**Testing**	**Classifier**
de Vos et al. (6)	✓	✓		1,554	1,036	CNN
Cano-Espinosa et al. (7)		✓		4,973	1,000	CNN
Lessmann et al. (8)		✓		1,181	506	CNN
Yang et al. (9)	✓		✓	32	40	SVM
Wolterink et al. (10)			✓	150	100	CNN
Wolterink et al. (11)	✓			384	570	RF
Shahzad et al. (12)	✓			209	157	kNN
Išgum et al. (13)		✓		337	231	kNN, SVM
Sánchez et al. (14)	✓			200	76	kNN
Liu et al. (15)	✓			^*^	31	SVM
Kurkure et al. (16)	✓			100	105	SVM
Brunner et al. (17)	✓			^*^	30	SVM
**(B) Non-calcified plaque**	**Detect**	**Characterize**		**Training**	**Testing**	**Classifier**
Kolossváry et al. (18)		✓		^*^	7	LC
Masuda et al. (19)		✓		^*^	78	BO
Zreik et al. (20)	✓	✓		98	65	CNN
Zhao et al. (21)	✓	✓		^*^	18	SVM
Jawaid et al. (22)	✓			^*^	32	SVM
Wei et al. (23)	✓			^*^	83	LC
Yamak et al. (24)		✓		-	3	E
Kelm et al. (25)	✓	✓		^*^	229	RF
Zuluaga et al. (26)	✓			1/13^†^	14/2	SVM

Check marks in (A) indicate detection (Detect) or characterization (Characterize) of plaque, check marks in (B) indicate analysis in dedicated non-contrast-enhanced calcium scoring CT (CSCT), chest CT (Chest CT) or coronary CT angiography (CCTA) images. The number of patients included for method development (Training) and evaluation (Testing) are listed,

* *indicates cross-validation and - indicates training on non-patient data. The classifier with which the primary result was obtained is indicated (Classifier, see section 2 for abbreviations)*.

† *A total of 15 scans was divided into training sets ranging from 1 to 13 and respective test sets comprised of the remaining scans*.

### 3.1. Calcified Plaque

Coronary artery calcification (CAC) quantification or *scoring* is typically performed in dedicated non-contrast-enhanced, ECG-triggered, calcium scoring CT images (CSCT). Using dedicated software, an expert identifies voxels with a density over 130 Hounsfield units (HU) in the coronary arteries. Identified CAC is then quantified according to its volume, density, or a combination of both (27). CAC cannot only be quantified in CSCT, but also in other kinds of CT images visualizing the heart, such as cardiac CT angiography (CCTA) and non-gated chest CT. Calcium scoring is not considered a difficult task for trained clinicians, but it is time-consuming when performed in large numbers of images. Hence, automatic ML-based methods have been proposed.

ML-based calcium scoring methods proposed prior to the advent of deep learning have focused on identification of CAC lesions among a large set of samples, i.e., groups of connected voxels above 130 HU. Samples are described with features such as size, shape, appearance and location to distinguish CAC from other candidate lesions such as calcifications in the aorta. Location features are of particular importance, as recognized by Liu et al. (15), Kurkure et al. (16), and Brunner et al. (17) who proposed a heart coordinate system. Similarly, Sánchez et al. (14) described candidate locations relative to anatomical landmarks. Išgum et al. (13) used multi-atlas registration to estimate the location of the coronary artery tree, while Shahzad et al. (12) and Wolterink et al. (11) estimated the location of three major coronary arteries for per-vessel calcium scoring. Yang et al. (9) extracted coronary artery centerlines in CCTA images and propagated these to CSCT images of the same patients to provide location features.

Deep learning-based methods have typically classified individual voxels instead of candidate lesions. Due to the extreme imbalance between numbers of CAC and background voxels in CT images, Wolterink et al. (10) proposed to use two CNNs, where one CNN identified candidate voxels in CCTA and the second CNN further discriminated among identified candidates. Similarly, Lessmann et al. (8) used two CNNs to identify calcified voxels in chest CT. Cano-Espinosa et al. (7) and de Vos et al. (6) avoid voxel-based classification altogether by directly regressing calcium scores in chest CT, enabling automatic scoring in less than a second.

Automatic CAC scoring methods have been validated in large data sets (28) and in other types of CT scans in which the heart is routinely visualized, such as attenuation correction images for PET-CT (29) and CT images acquired for radiotherapy treatment planning (30–32). Wolterink et al. presented a public data set with reference standard for standardized evaluation of CAC scoring in CSCT (33).

### 3.2. Non-calcified Plaque

Non-calcified plaque is typically lipid-rich and vulnerable to rupture, causing acute coronary syndrome (34). ML-based analysis methods in CCTA have been developed for detection or localization of non-calcified plaque, as well as characterization of lipid and fibrous plaque components.

Coronary artery localization by means of centerline extraction is a typical preprocessing step for ML-based plaque analysis. Traditionally, many automatic centerline extraction methods have been based on minimum cost paths between proximal and distal artery points (35, 36). ML has been used to verify automatic centerline extraction results with an RF (25) or CNN (37). Alternatively, centerlines can be iteratively extracted based on a single seed point. Wolterink et al. (38) showed how such a tracker can be guided by a 3D CNN that locally detects the artery orientation.

Coronary artery centerlines can be used to reconstruct CCTA volumes into images that allow better plaque visualization and identification. Zhao et al. (21), Jawaid et al. (22), Wei et al. (23), and Zuluaga et al. (26) used cross-sectional images along the coronary artery centerline to extract features describing the vessel wall shape and texture. In Jawaid et al. (22) and Wei et al. (23), these features were used in an SVM or linear classifier to determine whether the image contained non-calcified plaque. Similarly, Zuluaga et al. (26) used such features to train an SVM classifying lesion segments as either healthy or diseased, i.e., containing non-calcified or calcified plaque. Zhao et al. (21) trained an SVM to classify cross-sectional images as healthy or containing non-calcified, calcified, or mixed plaque. For the same task, Zreik et al. (20) trained a recurrent CNN that did not depend on hand-crafted feature extraction. Kelm et al. (25) used an RF classifier to classify whether non-calcified or calcified plaque was present along a coronary artery centerline segment.

Characterization of individual components in non-calcified plaque is a challenging task due to low-contrast boundaries between plaque components (39). Yamak et al. (24) exploited additional attenuation data provided by dual-energy CT to characterize plaque in manually determined regions of interest in axial slices. To validate their model in patient scans, manual CCTA annotations by an expert were used. However, obtaining reliable manual reference annotations for non-calcified plaque in CCTA is challenging. Kolossváry et al. (18) determined the reference standard in CCTA through registration of histology images to *ex-vivo* CCTA scans. Features were extracted for each cross-sectional image and lesions were classified into advanced or early stage atherosclerosis using a linear classifier. Alternatively, Masuda et al. (19) used an *in-vivo* IVUS-based reference standard to train a boosting classifier with histogram-based features distinguishing fibrous from lipid plaque in CCTA.

## 4. Coronary Stenosis Detection and Characterization

Non-invasive assessment of CAD-induced stenotic lesions in CT prior to invasive treatment may prevent unnecessary costs and complications (40). Therefore, CT images have long been used to assess the *anatomical* significance of lesions by a local measurement of luminal narrowing. However, determination of the *functional* significance of a lesion by taking physiology into account can better stratify patients in need of treatment (41). In this section, we review ML algorithms for the detection and quantification of anatomically (section 4.1) and functionally (section 4.2) significant stenosis. Reviewed papers are listed in Table 2.

**Table 2:** Publications related to (A) anatomically and (B) functionally significant stenosis detection.

	**Structure**		**Patients**		
	**Artery**	**Myocardium**	**Training**	**Testing**	**Classifier**
**(A) Anatomical significance**					
Lee et al. (42)	✓		412	136	CNN
Freiman et al. (43)	✓		^*^	90	CNN
Zreik et al. (20)	✓		98	65	CNN
Huang et al. (44)	✓		45	7	CNN
Kang et al. (45)	✓		^*^	42	SVM
Xiong et al. (46)		✓	^*^	140	BO
Mukhopadhyay et al. (47)		✓	^*^	27	ANN
Kelm et al. (25)	✓		^*^	229	RF
Zuluaga et al. (48)	✓		9	9	SVM
**(B) Functional significance**					
Kumamaru et al. (49)	✓		^*^	131	CNN
Wang et al. (50)	✓		8	63	ANN
Hae et al. (51)		✓	932	279	BO
Dey et al. (52)	✓		^*^	254	BO
Zreik et al. (53)		✓	^*^	166	SVM
Han et al. (54)		✓	^*^	252	BO
Itu et al. (55)	✓		-	87	ANN

*Check marks indicate arterial (Artery) or myocardial (Myocardium) analysis. The number of patients included for method development (Training) and evaluation (Testing) are listed*,

* *indicates cross-validation and - indicates training on non-patient data. The classifier with which the primary result was obtained is indicated (Classifier, see section 2 for abbreviations)*.

### 4.1. Anatomical Significance

Identification of anatomically significant stenotic lesions in CCTA, i.e., those lesions causing a luminal narrowing of at least 50%, allows a first assessment of the severity of stenosis in patients with symptoms of CAD. While this assessment is often based on visual estimation by a clinician, this is a difficult task (56) with substantial inter-observer variability (57). ML-based automatic approaches could reduce this variability.

Stenosis detection typically requires a local measurement of the lumen diameter and an estimation of the healthy lumen diameter. These estimates can be based on automatically extracted centerlines (section 3.2). Many centerline extraction methods also estimate the luminal radius at each centerline point, assuming a circular coronary artery profile (25, 38). However, circular artery profiles are not a realistic assumption for diseased vessel segments. Automatically extracted centerlines can also be used as an initialization for more detailed lumen segmentation. Huang et al. (44) used centerlines to obtain a reformatted image in which the lumen was segmented using a 3D CNN. Lee et al. (42) use centerlines to obtain a tube-shaped prior that is deformed to segment the coronary lumen.

Lumen segmentation is often considered a preprocessing step for stenosis detection, but it has been shown that stenosis degree can also be directly determined based on image data. Zuluaga et al. (48) detected stenosis and artery bifurcations with an SVM based on features obtained from concentric circles in cross-sectional images. Similarly, Kang et al. (45) used geometrical and plaque features in an SVM to detect obstructive lesions (> 50% narrowing) and non-obstructive lesions (25–50% narrowing). Zreik et al. (20) used a recurrent CNN to detect anatomically significant stenosis along the centerline. Freiman et al. (43) detected stenosis of at least intermediate severity (> 40% narrowing) using deep sparse autoencoders, a variation on CNNs.

Coronary stenoses are located in the arteries, but may restrict blood flow to myocardial segments. Mukhopadhyay et al. (47) used an ML approach to identify myocardial segments (58) affected by coronary stenosis. Hand-crafted feature vectors describing the endocardial surface shape were combined using a bag-of-words approach and classified with an ANN to identify affected segments. Xiong et al. (46) performed analysis of the full myocardium to detect existence of at least one anatomically significant stenosis. Instead of the shape of the endocardial surface, features in this approach described the attenuation and wall thickness of myocardial segments.

### 4.2. Functional Significance

The sensitivity of CCTA-based anatomical stenosis evaluation for detection of functionally significant stenosis is high when evaluated visually, but its specificity is moderate (41). The current reference standard for determination of functional significance of a stenosis is given by its fractional flow reserve (FFR), i.e., the ratio of flow distal of the stenosis to the flow proximal of the stenosis. FFR is measured invasively by inserting a special catheter in the coronary artery under hyperemic conditions. FFR below 0.80 indicates need for intervention (59). Treatment based on invasive FFR measurements can improve patient outcomes (59), but measurement of FFR is still relatively uncommon, which is due to associated cost and risk, as well as lack of vasodilator drugs (60).

FFR estimation based on CCTA scans (FFR_CT_) could provide reproducible physical measurement without the drawbacks of invasive procedures. FFR_CT_ has traditionally been based on computational fluid dynamics (CFD) (61, 62), i.e., numerical simulation of blood flow in a coronary tree model extracted from CCTA using lumen segmentation methods (section 4.1). These methods are accurate (63) but computationally expensive due to their iterative nature. This precludes their deployment on local workstations, and instead CFD simulations are typically performed on off-site dedicated systems. ML could be used to significantly speed up estimation of FFR_CT_.

Itu et al. (55) proposed an ANN model to predict an FFR value for each segment in the coronary artery tree, given local features based on the segment's geometry and global features based on the most severe stenoses. To train this model, a large data set of 12,000 synthetic coronary artery trees was generated and a reference standard was obtained through conventional CFD simulation. By only performing CFD simulations once in a training phase, the time required to perform FFR_CT_ was reduced by two orders of magnitude. The diagnostic value of this method has been demonstrated thoroughly (64–76). Yu et al. (77) further demonstrated additional prognostic value of CT morphological index for the method proposed by Itu et al. (55). Wang et al. (50) proposed to use a recurrent ANN that can model long-range dependencies between segments.

Both conventional CFD-based FFR_CT_ and the methods proposed in Wang et al. (50) and Itu et al. (55) are based only on the geometry of the coronary artery tree model, and are thus susceptible to errors by the segmentation method used to obtain this model (78). Instead, Dey et al. (52) proposed to combine geometric features with semi-automatically obtained plaque and attenuation gradient measurements to identify arteries with functionally significant stenosis. Other methods skip explicit coronary artery centerline extraction and lumen segmentation altogether. Kumamaru et al. (49) trained a CNN to extract a map showing the contrast-enhanced territories in CCTA and used this map in a classifier to predict the minimum FFR value in a patient. Alternatively, analysis can be moved from the cause—the coronary arteries—to the effect, i.e., the myocardium. Han et al. (54) subdivided the separated endocardium and epicardium into the American Heart Association (AHA) 17 segments (58), with 3 features per segment characterizing perfusion and wall thickness. However, the trained boosting classifier showed only moderate accuracy for patientwise prediction of abnormal FFR values. For the same purpose, Zreik et al. (53) trained an SVM based on features from myocardial regions extracted from CCTA. Clinical evaluation of this method yielded improved diagnostic accuracy of FFR_CT_ over visual evaluation of stenosis (79). Hae et al. (51) increased accuracy of FFR-prediction by including the tissue volume subtended to a stenotic lesion in analysis. However, determination of lesion position required additional analysis including artery tree segmentation.

## 5. Discussion

We have presented a survey of applications of ML for detection, characterization and quantification of atherosclerotic plaque and stenosis in cardiac CT. We found that while ML has been a mainstay of cardiac image analysis for years, the recent emergence of deep learning has accelerated progress in the field. Machine learning has the potential to unburden clinicians from time-consuming tasks and change diagnostic procedures, thereby reducing healthcare costs. Moreover, low-cost ML-based analysis could be added to screening studies as a secondary goal. In this survey, we have focused on ML for CAD analysis. For a broader scope the reader is referred to Al'Aref et al. (80), Litjens et al. (81), Nicol et al. (82), Petersen et al. (83), and Singh et al. (84).

We have reviewed plaque and stenosis analysis methods in separate sections, but formation of plaque and stenosis is naturally related and many papers have proposed simultaneous analysis [e.g., (48, 53)]. Moreover, (semi-)automatic identification of plaque or stenosis is often only an intermediate step for prediction of cardiovascular events. Motwani et al. (85) used stenosis scores and plaque characteristics to develop a model for 5 years all-cause mortality prediction. Similarly, Johnson et al. (86) showed that an ML model taking into account per segment coronary artery characteristics can outperform hand-crafted models for prediction of adverse cardiac events. Van Rosendael et al. (87) developed a model for all-cause mortality prediction in combination with future myocardial infarction based only on hand-crafted features derived from CCTA scans. Furthermore, some methods directly predict presence of CAD from medical images, i.e., chest CT (88) or non-contrast-enhanced cardiac CT (89). While these approaches only require one label per patient and large data sets are thus not expensive to obtain, the interpretability of predictions may be limited. Interpretability might constitute an opportunity, not only to improve reliability but also as it might increase medical knowledge by quantifying the diagnostic relevance of underlying phenomena.

The readiness of automatic analysis methods for clinical implementation depends on the complexity of the task, but also on other factors. ML algorithms require large training sets, and tasks with abundant data may be easier to automate. For example, obtaining a ground truth for e.g., non-calcified plaque characterization is very challenging. Therefore, data sets are generally small and ML algorithms remain at an early developmental stage. In contrast, large data sets are available for the development of ML-based CAC scoring methods, which has led to highly accurate results in both dedicated cardiac CT images (11) and other CT images visualizing the heart (8, 32). Similarly, ML-based FFR_CT_ development is aided by the availability of large data sets with CFD-derived reference values. An important remaining step toward clinical application of FFR_CT_ lies in performance evaluation specifically for subjects around the FFR threshold of 0.8, which were shown to be most challenging (90). Furthermore, a recent study showed that not all CCTA exams are suitable for FFR_CT_ analysis (78).

Many challenges in the adoption of machine learning methods in the clinic are not exclusive to CAD detection in cardiac CT. For example, ML algorithms could show unexpected behavior, motivating research into ML interpretability and explainability (91). Furthermore, it is important to point out that ML algorithms are often trained and evaluated on single center studies with high risk for selective biases, and under exclusion of low quality scans.

Despite these challenges, current rapid development allows for justifiable hope that the importance of ML algorithms in cardiac CT will not cease to increase in near future, with benefits for clinicians and patients alike.

## Author Contributions

NH and SV drafted the manuscript, which was critically revised and edited by JW, TL, and II. Authors agree to be accountable for all aspects of the work.

### Conflict of Interest

II and TL received institutional research projects by Dutch Technology Foundation cofunded by Pie Medical Imaging and Philips Healthcare (P15-26), the Netherlands Organisation for Health Research and Development with participation of Pie Medical Imaging (104003009). II received institutional research project by Dutch Technology Foundation cofunded by Pie Medical Imaging (12726). II and TL are cofounders and shareholders of Quantib-U BV. The remaining authors declare that the research was conducted in the absence of any commercial or financial relationships that could be construed as a potential conflict of interest.

## References

[1] Levin DC Parker L Halpern EJ Rao VM. Coronary CT angiography: reversal of earlier utilization trends. J Am Coll Radiol. (2019) 16:147–55. 10.1016/j.jacr.2018.07.022 30158087

[2] Dreisbach JG Nicol ED Roobottom CA Padley S Roditi G. Challenges in delivering computed tomography coronary angiography as the first-line test for stable chest pain. Heart. (2018) 104:921–7. 10.1136/heartjnl-2017-311846 29138258 PMC5969350

[3] Litjens G Kooi T Bejnordi BE Setio AAA Ciompi F Ghafoorian M. A survey on deep learning in medical image analysis. Med Image Anal. (2017) 42:60–88. 10.1016/j.media.2017.07.005 28778026

[4] Jordan MI Mitchell TM. Machine learning: trends, perspectives, and prospects. Science. (2015) 349:255–60. 10.1126/science.aaa8415 26185243

[5] Insull W. The pathology of atherosclerosis: plaque development and plaque responses to medical treatment. Am J Med. (2009) 122(1, Suppl.):S3–14. 10.1016/j.amjmed.2008.10.013 19110086

[6] de Vos BD Wolterink JM Leiner T Jong PAd Lessmann N Išgum I. Direct automatic coronary calcium scoring in cardiac and chest CT. IEEE Trans. Med. Imaging. (2019) 38:2127–38. 10.1109/TMI.2019.2899534 30794169

[7] Cano-Espinosa C González G Washko GR Cazorla M Estépar RSJ Automated agatston score computation in non-ECG gated CT scans using deep learning. In: Proceedings of SPIE–the International Society for Optical Engineering. vol. 10574 (2018). Available online at: https://www.ncbi.nlm.nih.gov/pmc/articles/PMC6095680/10.1117/12.2293681 PMC6095680 30122801

[8] Lessmann N Ginneken Bv Zreik M Jong PAd Vos BDd Viergever MA. Automatic calcium scoring in low-dose chest CT using deep neural networks with dilated convolutions. IEEE Trans Med Imaging. (2018) 37:615–25. 10.1109/TMI.2017.2769839 29408789

[9] Yang G Chen Y Ning X Sun Q Shu H Coatrieux JL. Automatic coronary calcium scoring using noncontrast and contrast CT images. Med Phys. (2016) 43:2174. 10.1118/1.4945045 27147329

[10] Wolterink JM Leiner T de Vos BD van Hamersvelt RW Viergever MA Išgum I. Automatic coronary artery calcium scoring in cardiac CT angiography using paired convolutional neural networks. Med Image Anal. (2016) 34:123–36. 10.1016/j.media.2016.04.004 27138584

[11] Wolterink JM Leiner T Takx RAP Viergever MA Išgum I. Automatic coronary calcium scoring in non-contrast-enhanced ECG-triggered cardiac CT with ambiguity detection. IEEE Trans Med Imaging. (2015) 34:1867–78. 10.1109/TMI.2015.2412651 25794387

[12] Shahzad R van Walsum T Schaap M Rossi A Klein S Weustink AC. Vessel specific coronary artery calcium scoring: an automatic system. Acad Radiol. (2013) 20:1–9. 10.1016/j.acra.2012.07.018 22981481

[13] Išgum I Prokop M Niemeijer M Viergever MA van Ginneken B. Automatic coronary calcium scoring in low-dose chest computed tomography. IEEE Trans Med Imaging. (2012) 31:2322–34. 10.1109/TMI.2012.2216889 22961297

[14] Sánchez CI Niemeijer M Išgum I Dumitrescu A Suttorp-Schulten MSA Abràmoff MD. Contextual computer-aided detection: improving bright lesion detection in retinal images and coronary calcification identification in CT scans. Med Image Anal. (2012) 16:50–62. 10.1016/j.media.2011.05.004 21689964

[15] Liu Q Qian Z Marvasty I Rinehart S Voros S Metaxas DN Lesion-specific coronary artery calcium quantification for predicting cardiac event with multiple instance support vector machines. In: Medical Image Computing and Computer-Assisted Intervention: MICCAI, Vol. 13 Beijing (2010). p. 484–92. 10.1007/978-3-642-15705-9_59 20879266

[16] Kurkure U Chittajallu DR Brunner G Le YH Kakadiaris IA. A supervised classification-based method for coronary calcium detection in non-contrast CT. Int J Cardiovasc Imaging. (2010) 26:817–28. 10.1007/s10554-010-9607-2 20229312

[17] Brunner G Chittajallu DR Kurkure U Kakadiaris IA. Toward the automatic detection of coronary artery calcification in non-contrast computed tomography data. Int J Cardiovasc Imaging. (2010) 26:829–38. 10.1007/s10554-010-9608-1 20232154

[18] Kolossváry M Karády J Kikuchi Y Ivanov A Schlett CL Lu MT. Radiomics versus visual and histogram-based assessment to identify atheromatous lesions at coronary CT angiography: an *ex vivo* study. Radiology. (2019) 293:190407. 10.1148/radiol.2019190407 31385755 PMC6776230

[19] Masuda T Nakaura T Funama Y Okimoto T Sato T Higaki T. Machine-learning integration of CT histogram analysis to evaluate the composition of atherosclerotic plaques: validation with IB-IVUS. J Cardiovasc Comput Tomogr. (2019) 13:163–9. 10.1016/j.jcct.2018.10.018 30529218

[20] Zreik M van Hamersvelt RW Wolterink JM Leiner T Viergever MA Išgum I. A recurrent CNN for automatic detection and classification of coronary artery plaque and stenosis in coronary CT angiography. IEEE Trans Med Imaging. (2019) 38:1588–98. 10.1109/TMI.2018.2883807 30507498

[21] Zhao F Wu B Chen F Cao X Yi H Hou Y. An automatic multi-class coronary atherosclerosis plaque detection and classification framework. Med Biol Eng Comput. (2019) 57:245–57. 10.1007/s11517-018-1880-6 30088125

[22] Jawaid MM Riaz A Rajani R Reyes-Aldasoro CC Slabaugh G. Framework for detection and localization of coronary non-calcified plaques in cardiac CTA using mean radial profiles. Comput Biol Med. (2017) 89:84–95. 10.1016/j.compbiomed.2017.07.021 28797740

[23] Wei J Zhou C Chan HP Chughtai A Agarwal P Kuriakose J. Computerized detection of noncalcified plaques in coronary CT angiography: evaluation of topological soft gradient prescreening method and luminal analysis. Med Phys. (2014) 41:081901. 10.1118/1.4885958 25086532 PMC4105962

[24] Yamak D Panse P Pavlicek W Boltz T Akay M. Non-calcified coronary atherosclerotic plaque characterization by dual energy computed tomography. IEEE J Biomed Health Informat. (2014) 18:939–45. 10.1109/JBHI.2013.2295534 24808227

[25] Kelm BM Mittal S Zheng Y Tsymbal A Bernhardt D Vega-Higuera F Detection, grading and classification of coronary stenoses in computed tomography angiography. In: Medical Image Computing and Computer-Assisted Intervention: MICCAI, Vol. 14 Toronto, ON (2011). p. 25–32. 10.1007/978-3-642-23626-6_4 22003680

[26] Zuluaga MA Hush D Delgado Leyton EJF Hernández Hoyos M Orkisz M Learning from only positive and unlabeled data to detect lesions in vascular CT images. In: Medical Image Computing and Computer-Assisted Intervention: MICCAI, Vol. 14 Toronto, ON (2011). p. 9–16. 10.1007/978-3-642-23626-6_2 22003678

[27] Agatston AS Janowitz WR Hildner FJ Zusmer NR Viamonte M Detrano R. Quantification of coronary artery calcium using ultrafast computed tomography. J Am Coll Cardiol. (1990) 15:827–32. 10.1016/0735-1097(90)90282-T 2407762

[28] Takx RAP de Jong PA Leiner T Oudkerk M de Koning HJ Mol CP. Automated coronary artery calcification scoring in non-gated chest CT: agreement and reliability. PLoS ONE. (2014) 9:e91239. 10.1371/journal.pone.0091239 24625525 PMC3953377

[29] Išgum I de Vos BD Wolterink JM Dey D Berman DS Rubeaux M Automatic determination of cardiovascular risk by CT attenuation correction maps in Rb-82 PET/CT. J Nucl Cardiol. (2018) 25:2133–42. 10.1007/s12350-017-0866-3 28378112 PMC5628109

[30] Emaus MJ Išgum I van Velzen SGM van den Bongard HJGD Gernaat SAM Lessmann N. Bragatston study protocol: a multicentre cohort study on automated quantification of cardiovascular calcifications on radiotherapy planning CT scans for cardiovascular risk prediction in patients with breast cancer. BMJ Open. (2019) 9:e028752. 10.1136/bmjopen-2018-028752 31352417 PMC6661654

[31] Gernaat SAM Išgum I de Vos BD Takx RAP Young-Afat DA Rijnberg N. Automatic coronary artery calcium scoring on radiotherapy planning CT scans of breast cancer patients: reproducibility and association with traditional cardiovascular risk factors. PLoS ONE. (2016) 11:e0167925. 10.1371/journal.pone.0167925 27936125 PMC5148008

[32] Gernaat SAM van Velzen SGM Koh V Emaus MJ Išgum I Lessmann N. Automatic quantification of calcifications in the coronary arteries and thoracic aorta on radiotherapy planning CT scans of Western and Asian breast cancer patients. Radiother Oncol. (2018) 127:487–92. 10.1016/j.radonc.2018.04.011 29703498

[33] Wolterink JM Leiner T de Vos BD Coatrieux JL Kelm BM Kondo S. An evaluation of automatic coronary artery calcium scoring methods with cardiac CT using the orCaScore framework. Med Phys. (2016) 43:2361. 10.1118/1.4945696 27147348

[34] Virmani R Burke AP Farb A Kolodgie FD. Pathology of the vulnerable plaque. J Am Coll Cardiol. (2006) 47(Suppl. 8):C13–18. 10.1016/j.jacc.2005.10.065 16631505

[35] Lesage D Angelini ED Bloch I Funka-Lea G. A review of 3D vessel lumen segmentation techniques: models, features and extraction schemes. Med Image Anal. (2009) 13:819–45. 10.1016/j.media.2009.07.011 19818675

[36] Schaap M Metz CT van Walsum T van der Giessen AG Weustink AC Mollet NR. Standardized evaluation methodology and reference database for evaluating coronary artery centerline extraction algorithms. Med Image Anal. (2009) 13:701–14. 10.1016/j.media.2009.06.003 19632885 PMC3843509

[37] Gülsün MA Funka-Lea G Sharma P Rapaka S Zheng Y Coronary centerline extraction via optimal flow paths and CNN path pruning. In: Ourselin S Joskowicz L Sabuncu MR Unal G Wells W editors. Medical Image Computing and Computer-Assisted Intervention: MICCAI. Lecture Notes in Computer Science. Athens: Springer International Publishing (2016). p. 317–25.

[38] Wolterink JM van Hamersvelt RW Viergever MA Leiner T Išgum I. Coronary artery centerline extraction in cardiac CT angiography using a CNN-based orientation classifier. Med Image Anal. (2019) 51:46–60. 10.1016/j.media.2018.10.005 30388501

[39] Kristanto W van Ooijen PMA Jansen-van der Weide MC Vliegenthart R Oudkerk M. A meta analysis and hierarchical classification of HU-based atherosclerotic plaque characterization criteria. PLoS ONE. (2013) 8:e73460. 10.1371/journal.pone.0073460 24019924 PMC3760884

[40] Pijls NHJ Tanaka N Fearon WF. Functional assessment of coronary stenoses: can we live without it? Eur Heart J. (2013) 34:1335–44. 10.1093/eurheartj/ehs436 23257950

[41] Meijboom WB Van Mieghem CAG van Pelt N Weustink A Pugliese F Mollet NR. Comprehensive assessment of coronary artery stenoses: computed tomography coronary angiography versus conventional coronary angiography and correlation with fractional flow reserve in patients with stable angina. J Am Coll Cardiol. (2008) 52:636–43. 10.1016/j.jacc.2008.05.024 18702967

[42] Lee MCH Petersen K Pawlowski N Glocker B Schaap M. TETRIS: template transformer networks for image segmentation with shape priors. IEEE Trans Med Imaging. (2019) 38:2596–606. 10.1109/TMI.2019.2905990 30908196

[43] Freiman M Manjeshwar R Goshen L. Unsupervised abnormality detection through mixed structure regularization (MSR) in deep sparse autoencoders. Med Phys. (2019) 46:2223–31. 10.1002/mp.13464 30821364

[44] Huang W Huang L Lin Z Huang S Chi Y Zhou J. Coronary artery segmentation by deep learning neural networks on computed tomographic coronary angiographic images. In: Conference Proceedings: Annual International Conference of the IEEE Engineering in Medicine and Biology Society. IEEE Engineering in Medicine and Biology Society. Annual Conference, Vol. 2018. Honolulu, HI (2018). p. 608–11. 30440470 10.1109/EMBC.2018.8512328

[45] Kang D Dey D Slomka PJ Arsanjani R Nakazato R Ko H. Structured learning algorithm for detection of nonobstructive and obstructive coronary plaque lesions from computed tomography angiography. J Med Imaging. (2015) 2:014003. 10.1117/1.JMI.2.1.014003 26158081 PMC4478984

[46] Xiong G Kola D Heo R Elmore K Cho I Min JK. Myocardial perfusion analysis in cardiac computed tomography angiographic images at rest. Med Image Anal. (2015) 24:77–89. 10.1016/j.media.2015.05.010 26073787 PMC4536577

[47] Mukhopadhyay A Qian Z Bhandarkar SM Liu T Rinehart S Voros S. Morphological analysis of the left ventricular endocardial surface and its clinical implications. In: Medical Image Computing and Computer-Assisted Intervention: MICCAI, Vol. 15. Nice (2012). p. 502–10. 23286086 10.1007/978-3-642-33418-4_62

[48] Zuluaga MA Magnin IE Hernández Hoyos M Delgado Leyton EJF Lozano F Orkisz M. Automatic detection of abnormal vascular cross-sections based on density level detection and support vector machines. Int J Comput Assist Radiol Surg. (2011) 6:163–74. 10.1007/s11548-010-0494-8 20549375

[49] Kumamaru KK Fujimoto S Otsuka Y Kawasaki T Kawaguchi Y Kato E. Diagnostic accuracy of 3D deep-learning-based fully automated estimation of patient-level minimum fractional flow reserve from coronary computed tomography angiography. Eur Heart J Cardiovasc Imaging. (2019). 10.1093/ehjci/jez160. [Epub ahead of print]. 31230076

[50] Wang ZQ Zhou YJ Zhao YX Shi DM Liu YY Liu W. Diagnostic accuracy of a deep learning approach to calculate FFR from coronary CT angiography. J Geriatr Cardiol. (2019) 16:42–8. 10.11909/j.issn.1671-5411.2019.01.010 30800150 PMC6379239

[51] Hae H Kang SJ Kim WJ Choi SY Lee JG Bae Y. Machine learning assessment of myocardial ischemia using angiography: development and retrospective validation. PLoS Med. (2018) 15:e1002693. 10.1371/journal.pmed.1002693 30422987 PMC6233920

[52] Dey D Gaur S Ovrehus KA Slomka PJ Betancur J Goeller M Integrated prediction of lesion-specific ischemia from quantitative coronary CT Angiography using machine learning: a multicenter study. Eur Radiol. (2018) 28:2655–64. 10.1007/s00330-017-5223-z 29352380 PMC5940537

[53] Zreik M Lessmann N van Hamersvelt RW Wolterink JM Voskuil M Viergever MA. Deep learning analysis of the myocardium in coronary CT angiography for identification of patients with functionally significant coronary artery stenosis. Med Image Anal. (2018) 44:72–85. 10.1016/j.media.2017.11.008 29197253

[54] Han D Lee JH Rizvi A Gransar H Baskaran L Schulman-Marcus J. Incremental role of resting myocardial computed tomography perfusion for predicting physiologically significant coronary artery disease: a machine learning approach. J Nucl Cardiol. (2018) 25:223–33. 10.1007/s12350-017-0834-y 28303473

[55] Itu L Rapaka S Passerini T Georgescu B Schwemmer C Schoebinger M. A machine-learning approach for computation of fractional flow reserve from coronary computed tomography. J Appl Physiol. (2016) 121:42–52. 10.1152/japplphysiol.00752.2015 27079692

[56] Pugliese F Hunink MGM Gruszczynska K Alberghina F Malagó R van Pelt N. Learning curve for coronary CT angiography: what constitutes sufficient training? Radiology. (2009) 251:359–68. 10.1148/radiol.2512080384 19401570

[57] Arbab-Zadeh A Hoe J. Quantification of coronary arterial stenoses by multidetector CT angiography in comparison with conventional angiography methods, caveats, and implications. JACC Cardiovasc Imaging. (2011) 4:191–202. 10.1016/j.jcmg.2010.10.011 21329905

[58] Cerqueira MD Weissman NJ Dilsizian V Jacobs AK Kaul S Laskey WK. Standardized myocardial segmentation and nomenclature for tomographic imaging of the heart. A statement for healthcare professionals from the Cardiac Imaging Committee of the Council on Clinical Cardiology of the American Heart Association. Circulation. (2002) 105:539–42. 10.1161/hc0402.102975 11815441

[59] Tonino PAL De Bruyne B Pijls NHJ Siebert U Ikeno F van' t Veer M. Fractional flow reserve versus angiography for guiding percutaneous coronary intervention. N Engl J Med. (2009) 360:213–24. 10.1056/NEJMoa0807611 19144937

[60] Petraco R Park JJ Sen S Nijjer SS Malik IS Echavarría-Pinto M. Hybrid iFR-FFR decision-making strategy: implications for enhancing universal adoption of physiology-guided coronary revascularisation. EuroIntervention. (2013) 8:1157–65. 10.4244/EIJV8I10A179 23256988

[61] Taylor CA Fonte TA Min JK. Computational fluid dynamics applied to cardiac computed tomography for noninvasive quantification of fractional flow reserve: scientific basis. J Am Coll Cardiol. (2013) 61:2233–41. 10.1016/j.jacc.2012.11.083 23562923

[62] Tesche C Vliegenthart R Duguay TM De Cecco CN Albrecht MH De Santis D. Coronary computed tomographic angiography-derived fractional flow reserve for therapeutic decision making. Am J Cardiol. (2017) 120:2121–7. 10.1016/j.amjcard.2017.08.034 29102036

[63] Nørgaard BL Leipsic J Gaur S Seneviratne S Ko BS Ito H. Diagnostic performance of noninvasive fractional flow reserve derived from coronary computed tomography angiography in suspected coronary artery disease: the NXT trial (Analysis of Coronary Blood Flow Using CT Angiography: next steps). J Am Coll Cardiol. (2014) 63:1145–55. 10.1016/j.jacc.2013.11.043 24486266

[64] Baumann S Renker M Schoepf UJ De Cecco CN Coenen A De Geer J. Gender differences in the diagnostic performance of machine learning coronary CT angiography-derived fractional flow reserve -results from the MACHINE registry. Eur J Radiol. (2019) 119:108657. 10.1016/j.ejrad.2019.108657 31521876

[65] Coenen A Kim YH Kruk M Tesche C De Geer J Kurata A. Diagnostic accuracy of a machine-learning approach to coronary computed tomographic angiography-based fractional flow reserve: result from the MACHINE consortium. Circulat Cardiovasc Imaging. (2018) 11:e007217. 10.1161/CIRCIMAGING.117.007217 29914866

[66] Duguay TM Tesche C Vliegenthart R De Cecco CN Lin H Albrecht MH. Coronary computed tomographic angiography-derived fractional flow reserve based on machine learning for risk stratification of non-culprit coronary narrowings in patients with acute coronary syndrome. Am J Cardiol. (2017) 120:1260–6. 10.1016/j.amjcard.2017.07.008 28844517

[67] Hu X Yang M Han L Du Y. Diagnostic performance of machine-learning-based computed fractional flow reserve (FFR) derived from coronary computed tomography angiography for the assessment of myocardial ischemia verified by invasive FFR. Int J Cardiovasc Imaging. (2018) 34:1987–996. 10.1007/s10554-018-1419-9 30062537

[68] Kurata A Fukuyama N Hirai K Kawaguchi N Tanabe Y Okayama H. On-site computed tomography-derived fractional flow reserve using a machine-learning algorithm - clinical effectiveness in a retrospective multicenter cohort. Circulat J. (2019) 83:1563–71. 10.1253/circj.CJ-19-0163 31178524

[69] Nous FMA Coenen A Boersma E Kim YH Kruk MBP Tesche C. Comparison of the diagnostic performance of coronary computed tomography angiography-derived fractional flow reserve in patients with versus without diabetes mellitus (from the MACHINE consortium). Am J Cardiol. (2019) 123:537–43. 10.1016/j.amjcard.2018.11.024 30553510

[70] Tang CX Wang YN Zhou F Schoepf UJ Assen Mv Stroud RE. Diagnostic performance of fractional flow reserve derived from coronary CT angiography for detection of lesion-specific ischemia: a multi-center study and meta-analysis. Eur J Radiol. (2019) 116:90–7. 10.1016/j.ejrad.2019.04.011 31153580

[71] Tesche C De Cecco CN Albrecht MH Duguay TM Bayer RR Litwin SE. Coronary CT angiography-derived fractional flow reserve. Radiology. (2017) 285:17–33. 10.1148/radiol.2017162641 28926310

[72] Tesche C Otani K De Cecco CN Coenen A De Geer J Kruk M. Influence of coronary calcium on diagnostic performance of machine learning CT-FFR: results from MACHINE registry. JACC Cardiovasc Imaging. (2019). 10.1016/j.jcmg.2019.06.027 31422141

[73] von Knebel Doeberitz PL De Cecco CN Schoepf UJ Duguay TM Albrecht MH van Assen M. Coronary CT angiography-derived plaque quantification with artificial intelligence CT fractional flow reserve for the identification of lesion-specific ischemia. Eur Radiol. (2019) 29:2378–87. 10.1007/s00330-018-5834-z 30523456

[74] Wardziak L Kruk M Pleban W Demkow M Ruzyllo W Dzielinska Z. Coronary CTA enhanced with CTA based FFR analysis provides higher diagnostic value than invasive coronary angiography in patients with intermediate coronary stenosis. J Cardiovasc Comput Tomogr. (2019) 13:62–7. 10.1016/j.jcct.2018.10.004 30309764

[75] Yu M Lu Z Shen C Yan J Wang Y Lu B The best predictor of ischemic coronary stenosis: subtended myocardial volume, machine learning-based FFRCT, or high-risk plaque features? Eur. Radiol. (2019) 29:3647–57. 10.1007/s00330-019-06139-2 30903334

[76] Zhou F Tang CX Schoepf UJ Tesche C Rollins JD Liu H. Machine learning using CT-FFR predicts proximal atherosclerotic plaque formation associated with LAD myocardial bridging. JACC Cardiovasc Imaging. (2019) 12:1591–3. 10.1016/j.jcmg.2019.01.018 30878419

[77] Yu M Lu Z Li W Wei M Yan J Zhang J. CT morphological index provides incremental value to machine learning based CT-FFR for predicting hemodynamically significant coronary stenosis. Int J Cardiol. (2018) 265:256–61. 10.1016/j.ijcard.2018.01.075 29885695

[78] Pontone G Weir-McCall JR Baggiano A Del Torto A Fusini L Guglielmo M. Determinants of rejection rate for coronary CT angiography fractional flow reserve analysis. Radiology. (2019) 292:597–605. 10.1148/radiol.2019182673 31335283

[79] van Hamersvelt RW Zreik M Voskuil M Viergever MA Išgum I Leiner T. Deep learning analysis of left ventricular myocardium in CT angiographic intermediate-degree coronary stenosis improves the diagnostic accuracy for identification of functionally significant stenosis. Eur Radiol. (2019) 29:2350–9. 10.1007/s00330-018-5822-3 30421020 PMC6443613

[80] Al'Aref SJ Anchouche K Singh G Slomka PJ Kolli KK Kumar A. Clinical applications of machine learning in cardiovascular disease and its relevance to cardiac imaging. Eur Heart J. (2019) 40:1975–86. 10.1093/eurheartj/ehy404 30060039

[81] Litjens G Ciompi F Wolterink JM de Vos BD Leiner T Teuwen J. State-of-the-art deep learning in cardiovascular image analysis. JACC Cardiovasc Imaging. (2019) 12(8 Pt 1):1549–65. 10.1016/j.jcmg.2019.06.009 31395244

[82] Nicol ED Norgaard BL Blanke P Ahmadi A Weir-McCall J Horvat PM. The future of cardiovascular computed tomography: advanced analytics and clinical insights. JACC. (2019) 12:1058–72. 10.1016/j.jcmg.2018.11.037 31171259

[83] Petersen SE Abdulkareem M Leiner T. Artificial intelligence will transform cardiac imaging-opportunities and challenges. Front Cardiovasc Med. (2019) 6:133. 10.3389/fcvm.2019.00133 31552275 PMC6746883

[84] Singh G Al'Aref SJ Van Assen M Kim TS van Rosendael A Kolli KK. Machine learning in cardiac CT: basic concepts and contemporary data. J Cardiovasc Comput Tomogr. (2018) 12:192–201. 10.1016/j.jcct.2018.04.010 29754806

[85] Motwani M Dey D Berman DS Germano G Achenbach S Al-Mallah MH. Machine learning for prediction of all-cause mortality in patients with suspected coronary artery disease: a 5-year multicentre prospective registry analysis. Eur Heart J. (2017) 38:500–7. 10.1093/eurheartj/ehw188 27252451 PMC5897836

[86] Johnson KM Johnson HE Zhao Y Dowe DA Staib LH. Scoring of coronary artery disease characteristics on coronary CT angiograms by using machine learning. Radiology. (2019) 292:354–62. 10.1148/radiol.2019182061 31237495

[87] Van Rosendael AR Maliakal G Kolli KK Beecy A Al'Aref SJ Dwivedi A. Maximization of the usage of coronary CTA derived plaque information using a machine learning based algorithm to improve risk stratification; insights from the CONFIRM registry. J Cardiovasc Comput Tomogr. (2018) 12:204–9. 10.1016/j.jcct.2018.04.011 29753765

[88] Dormer JD Halicek M Ma L Reilly CM Schreibmann E Fei B Convolutional neural networks for the detection of diseased hearts using CT images and left atrium patches. In: Proceedings of SPIE–the International Society for Optical Engineering, Vol. 10575 San Diego, CA (2018). 10.1117/12.2293548 PMC6123226 30197463

[89] Mannil M von Spiczak J Manka R Alkadhi H. Texture analysis and machine learning for detecting myocardial infarction in noncontrast low-dose computed tomography: unveiling the invisible. Investigat Radiol. (2018) 53:338–43. 10.1097/RLI.0000000000000448 29420321

[90] Cook CM Petraco R Shun-Shin MJ Ahmad Y Nijjer S Al-Lamee R. Diagnostic accuracy of computed tomography-derived fractional flow reserve: a systematic review. JAMA Cardiol. (2017) 2:803–10. 10.1001/jamacardio.2017.1314 28538960

[91] Samek W Montavon G Vedaldi A Hansen LK Muller KR editors. Explainable AI: interpreting, explaining and visualizing deep learning. In: Lecture Notes in Artificial Intelligence, Lecture Notes ComputerState-of-the-Art Surveys. Springer International Publishing (2019). Available online at: https://www.springer.com/gp/book/9783030289539

