# Supplementary material for: Machine Learning for Assessment of Coronary Artery Disease in Cardiac CT: A Survey
Source: Front Cardiovasc Med. 2019 Nov 26;6:172. doi: 10.3389/fcvm.2019.00172 (PMC6988816; doi:10.3389/fcvm.2019.00172)
Supplement: Supplementary file 1 [file Data_Sheet_1.PDF]

Supplementary Material

1 SEARCH PROCESS

We searched PubMed for articles related to machine learning in cardiac computed tomography (CT) published within the last 10 years. For this purpose we constructed three semantically motivated search groups. For a positive search hit, at least one of the search terms within each of these groups had to be present in title or abstract. Group 1 constrained papers to the cardiac field, group 2 specified the imaging modality as CT and group 3 was directed towards Machine Learning methods. Specific search terms can be found in Table S1, yielding a total of 628 articles. Subsequently, papers were sorted into application focused categories, namely detection and characterization of plaque, detection and characterization of stenosis and prediction of outcome. Publications not matching one of these categories were discarded, resulting in a final selection of 59 papers. These papers were further stratified into primary papers, with focus on image processing methodology, and secondary papers, concerned with clinical evaluation of previously developed methods.

Table S1. Terms for search in PubMed. Three separate groups were specified, which were connected by the operator "AND". Terms within each group were connected by the operator "OR". If multiple words form a term, the exact combination had to appear. Asterisks denote wild card search, i.e. searching for any word which can be constructed from the given root.

cardiac field	imaging modality CT	machine learning methods
calcium	CT	machine learning
calcification*	CTs	deep learning
cardia*	computed tomograph*	artificial intelligence
coronary		computer vision
heart*		computer-aided diagnosis
FFR		neural network*
fractional flow reserve		convnet*
ischemia		CNN*
stenosis		deep architecture*
atrial		autoencoder*
plaque*		pattern recognition*
valve*		classifier*
infarction*		automatic detection
ventricle*		
